# *Plasmodium falciparum* genetic diversity and multiplicity of infection based on *msp-1*, *msp-2*, *glurp* and microsatellite genetic markers in sub-Saharan Africa: a systematic review and meta-analysis

**DOI:** 10.1186/s12936-024-04925-y

**Published:** 2024-04-08

**Authors:** Alex Mwesigwa, Moses Ocan, Benson Musinguzi, Rachel Wangi Nante, Joaniter I. Nankabirwa, Steven M. Kiwuwa, Alison Annet Kinengyere, Barbara Castelnuovo, Charles Karamagi, Ekwaro A. Obuku, Samuel L. Nsobya, Sam M. Mbulaiteye, Pauline Byakika-Kibwika

**Affiliations:** 1 Clinical Epidemiology Unit, School of Medicine, College of Health Sciences, Makerere University, P. O. Box 7072, Kampala, Uganda; 2 Department of Microbiology and Immunology, School of Medicine, Kabale University, P. O Box 314, Kabale, Uganda; 3 Department of Medicine, School of Medicine, College of Health Sciences, Makerere University, P. O. Box 7072, Kampala, Uganda; 4 Department of Pharmacology and Therapeutics, School of Biomedical Sciences, College of Health Sciences, Makerere University, P.O. Box 7072, Kampala, Uganda; 5 Department of Biochemistry, School of Biomedical Sciences, College of Health Sciences, Makerere, University, P.O. Box 7072, Kampala, Uganda; 6 Infectious Diseases Research Collaboration, College of Health Sciences, Makerere University, P.O. Box 7072, Kampala, Uganda; 7 Infectious Diseases Institute, College of Health Sciences, Makerere University, P. O. Box 7072, Kampala, Uganda; 8 Albert Cook Library, College of Health Sciences, Makerere University, P.O. Box 7072, Kampala, Uganda; 9 Division of Cancer Epidemiology and Genetics, National Cancer Institute, 9609 Medical Center Dr, 6E-118, Bethesda, MD 20892 USA; 10 Departent of Medical Laboratory Science, Faculty of Health Sciences, Muni University, P.O Box 725, Arua, Uganda; 11 African Center for Systematic Reviews and Knowledge Translation, College of Health Sciences, Makerere University, P.O. Box 7072, Kampala, Uganda; 12 Faculty of Epidemiology and Population Health, London School of Hygiene and Tropical Medicine, London, UK

**Keywords:** *Plasmodium falciparum*, Genetic diversity and multiplicity of infection, Sub-Saharan Africa

## Abstract

**Background:**

In sub-Saharan Africa (SSA), *Plasmodium falciparum* causes most of the malaria cases. Despite its crucial roles in disease severity and drug resistance, comprehensive data on *Plasmodium falciparum* genetic diversity and multiplicity of infection (MOI) are sparse in SSA. This study summarizes available information on genetic diversity and MOI, focusing on key markers (*msp-1, msp-2, glurp*, and microsatellites). The systematic review aimed to evaluate their influence on malaria transmission dynamics and offer insights for enhancing malaria control measures in SSA.

**Methods:**

The review was conducted following the Preferred Reporting Items for Systematic Review and Meta-Analysis (PRISMA) guidelines. Two reviewers conducted article screening, assessed the risk of bias (RoB), and performed data abstraction. Meta-analysis was performed using the random-effects model in STATA version 17.

**Results:**

The review included 52 articles: 39 cross-sectional studies and 13 Randomized Controlled Trial (RCT)/cohort studies, involving 11,640 genotyped parasite isolates from 23 SSA countries. The overall pooled mean expected heterozygosity was 0.65 (95% CI: 0.51–0.78). Regionally, values varied: East (0.58), Central (0.84), Southern (0.74), and West Africa (0.69). Overall pooled allele frequencies of *msp-1* alleles K1, MAD20, and RO33 were 61%, 44%, and 40%, respectively, while *msp-2* I/C 3D7 and FC27 alleles were 61% and 55%. Central Africa reported higher frequencies (K1: 74%, MAD20: 51%, RO33: 48%) than East Africa (K1: 46%, MAD20: 42%, RO33: 31%). For *msp-2*, East Africa had 60% and 55% for I/C 3D7 and FC27 alleles, while West Africa had 62% and 50%, respectively. The pooled allele frequency for *glurp* was 66%. The overall pooled mean MOI was 2.09 (95% CI: 1.88–2.30), with regional variations: East (2.05), Central (2.37), Southern (2.16), and West Africa (1.96). The overall prevalence of polyclonal *Plasmodium falciparum* infections was 63% (95% CI: 56–70), with regional prevalences as follows: East (62%), West (61%), Central (65%), and South Africa (71%).

**Conclusion:**

The study shows substantial regional variation in *Plasmodium falciparum* parasite genetic diversity and MOI in SSA. These findings suggest a need for malaria control strategies and surveillance efforts considering regional-specific factors underlying *Plasmodium falciparum* infection.

**Supplementary Information:**

The online version contains supplementary material available at 10.1186/s12936-024-04925-y.

## Background

*Plasmodium falciparum* presents a significant public health challenge in sub-Saharan Africa (SSA), constituting the majority of reported malaria cases. In 2022, out of the 249 million malaria cases recorded globally, 233 million occurred in SSA, contributing to an estimated 580,000 out of the 608,000 malaria-related deaths worldwide [1]. While the development of a robust immune response is necessary for controlling *Plasmodium falciparum* infection [2], timely diagnosis and the administration of effective treatments [3] are required to control symptomatic infection and reduce transmission.

The control of *Plasmodium falciparum* is hindered by the high propensity for genetic diversity of parasites infecting individuals and the frequency of multiplicity of infection (MOI) within individual infections. These factors favour immune evasion, may contribute to malaria pathology, and could promote the emergence of variants resistant to anti-malarial drugs [4]. Moreover, genetic diversity, particularly involving protein-coding genes targeted by diagnostic tests such as histidine-rich protein 2/3 (HRP2/3) [5], which have become important tools for malaria diagnosis and surveillance, could have significant implications for malaria surveillance and control.

Genetic diversity and MOI are emerging as relevant biomarkers of *Plasmodium falciparum* transmission. *Plasmodium falciparum* genetic diversity arises from genetic recombination during the parasite lifecycle in the mosquito [6], while MOI results from infection by multiple distinct parasite genotypes [7]. Infection by distinct parasite genotypes occurs either when an individual is bitten by different mosquitoes carrying unique parasite strains (superinfection) or when bitten by a single mosquito carrying multiple distinct genotypes (co-transmission) [8, 9].

The genetic diversity and MOI of *Plasmodium falciparum* may be assessed by targeted genotyping of markers such as *msp-1, msp-2*, and *glurp*, which are coding and therefore targets for immune evasion [10] or microsatellite markers, which are not targets for immune evasion [11]. High-throughput methods, including molecular (DNA) barcodes, targeted deep sequencing, and genome-wide variation analysis, have also been utilized [12, 13], but these are expensive. Although more labour-intensive and subject to some biases, such as amplification efficiency bias due to size differences between *msp-1, msp-2*, and *glurp* alleles [14, 15], genotyping of these markers is cheaper and more readily available in resource-limited settings in SSA [12].

The mean values of parasite genetic diversity and MOI are higher in areas with high malaria transmission intensity [16, 17] and lower in those with low transmission intensity [18]. Additionally, mean *Plasmodium falciparum* genetic diversity and MOI apparently decreased following the suppression of *Plasmodium falciparum* transmission intensity in areas of Ethiopia [19] and Senegal [20]. In other studies, mean values of *Plasmodium falciparum* genetic diversity were higher among individuals with symptomatic infections [21, 22] and lower in those with asymptomatic infections [23], and were inversely correlated with parasite density and patient age [24].

Data on *Plasmodium falciparum* genetic diversity and MOI are relatively sparse, making it difficult to easily identify relevant patterns in SSA. Some studies have focused solely on MOI but not genetic diversity [25, 26], while others have been conducted within a single country [27], or utilized a single genetic marker [28]. This study collated published data on *Plasmodium falciparum* genetic diversity and MOI in SSA and summarized this data for symptomatic and asymptomatic individuals using a few genetic markers that are widely utilised for parasite genotyping in SSA. The aim of the study was to generate a systematic summary that can inform public health initiatives for malaria control in different regions of SSA.

## Methods

### Study design and protocol registration

The systematic review was conducted using the Preferred Reporting Items for Systematic Review and Meta-Analysis (PRISMA) guidelines [29]. The review protocol is registered in PROSPERO (#CRD42021267661).

### Review question

The study reviewed data on *Plasmodium falciparum* genetic diversity and MOI in SSA based on *msp-1, msp-2, glurp*, and microsatellite genetic markers from articles published from January 2000 through May 2023. This period was chosen because access to malaria genomic technologies was reasonably high, yielding representative data in the regions sampled [30]. Also, the period coincides with rapid decline in malaria incidence in SSA [31]. The objectives of the review were to: a) characterize the geographical distribution of *Plasmodium falciparum* genetic diversity in SSA; b) determine the prevalence of *Plasmodium falciparum* polyclonal infections in SSA; and c) identify factors associated with *Plasmodium falciparum* genetic diversity and MOI in SSA.

### Search strategy and information sources

A systematic search, conducted by an experienced librarian (AAK), utilized PubMed, EMBASE, EBSCOhost, Web of Science, and the first 50 pages of Google Scholar after searching several pages and found no more relevant studies Additionally, citation lists of the identified articles were searched for additional relevant articles [32–37]. The search terms included keywords such as *'Plasmodium falciparum*,' '*P. falciparum* genetic diversity,' '*P. falciparum* multiplicity of infection,' and 'sub-Saharan Africa' (Additional file 1).

### Eligibility

#### Inclusion criteria

The review considered:

- Articles published in English,
- Study design, i.e., observational (cross-sectional/survey, case control, and cohort) or randomized clinical trials (RCTs),
- Minimum required data elements: country, sample size, calendar year(s) when the study was conducted, and detailed laboratory methods used to genotype markers for genetic diversity or MOI,
- Detailed methods for determining *Plasmodium falciparum* genetic diversity and MOI, including mean expected heterozygosity, allele frequencies, and mean MOI or percentage of multiple infections.

#### Exclusion criteria

- Absence of key terms ‘*Plasmodium falciparum* genetic diversity and or MOI’ in the title and or abstract,
- Studies using experimental animals,
- Review articles, case reports, case series, or editorials
- Use of inappropriate laboratory molecular methods (DNA extraction, PCR and then fragment analysis).

### Article screening and data extraction

The articles were deduplicated using Endnote software *version* X9. Subsequently, the unique articles underwent screening by two independent reviewers (AM and RWN), who also performed data abstraction using predetermined review criteria (Additional file 2). Abstracted data were compared, and any disagreements were resolved through discussion.

Harmonized extracted data included:

- Study characteristics (author name, article title, publication year, country, malaria transmission setting, and study design); participant characteristics (sample size, age group, and malaria clinical category),
- Malaria diagnosis and genotyping (malaria diagnosis method, name of genotyped markers, and PCR fragment analysis method),
- Outcome results on *Plasmodium falciparum* genetic diversity and MOI based on mean expected heterozygosity, allele frequencies of selected genotyped genetic markers, and the prevalence of polyclonal infections or mean MOI, respectively. Data on factors associated with Plasmodium falciparum genetic diversity and MOI were also extracted.

### Data analysis

Meta-analysis was performed using the random effects model (DerSimonian and Laird approach) in STATA (version 17, Stata Corporation, College Station, TX). Forest plots included only studies that reported measures of dispersion such as SD or CI for the respective effect sizes (Mean MOI and or Mean He) to enable computation of standard error for use in meta-analysis using STATA. Pooled estimates for *Plasmodium falciparum* genetic diversity and MOI were generated, sorted by region. Additionally, patterns of *Plasmodium falciparum* genetic diversity and MOI were assessed according to malaria clinical categories (asymptomatic and symptomatic malaria infection) [38] and specific genetic marker(s) used to evaluate *Plasmodium falciparum* genetic diversity and MOI [12].

### Heterogeneity analysis

Heterogeneity across the studies was assessed using the chi-squared test and Cochran’s Q statistic, with a 5% level of statistical significance [39], and the I-squared (I^2^) statistic [40]. An I^2^ statistic of 25% indicates low heterogeneity, 50% indicates moderate heterogeneity, and > 75% indicates high heterogeneity [41].

### Risk of bias and quality of evidence assessment

Risk of bias (RoB) in the selected articles was independently evaluated by two reviewers (AM, RWN) using an RoB assessment tool adapted from Joanna Briggs Institute’s (JBI) critical appraisal tools [42]. The quality of evidence was determined by two independent reviewers using the Grading of Recommendations, Assessment, Development, and Evaluations (GRADE) guidelines [43]. RoB assessment covered five domains: study design and limitations, inconsistency in selected articles, indirectness of the evidence, imprecision, and publication bias. Studies scoring 0 to 1, 2 to 3, 4 to 5, and at least 6 were judged to be very low, low, moderate, and high-quality studies, respectively.

### Publication bias

Publication bias was assessed by visualizing the asymmetry of the funnel plot and examining the presence and distribution of dots in the plot [44]. Egger’s statistical test was performed to assess the asymmetry of the funnel plot. A statistically significant result (*p* < 0.05) in Egger’s test indicates that the funnel plot asymmetry is due to small-study effects [45]. All data analysis was conducted using STATA *version* 17 software package (Stata Corporation, College Station, TX).

### Missing data

Variables that were missing from included articles were recorded as not reported (NR). Authors of articles with missing data were contacted for additional information, but only a small number (5/12: 41.67%) responded.

### Ethics considerations

The study used already published literature with no direct human subject contact and posed no risk to the participants who participated in the primary studies as determined by the Makerere University School of Medicine Institutional Review Board (# Mak-SOMREC-2021-152) and Uganda National Council for Science and Technology (# HS2744ES) (Table 1).

**Table 1:** The PECOST framework

Element	Description
Population	Individuals infected with *P. falciparum*
Exposure	*P. falciparum* infection
Comparator	None
Outcomes	Primary outcomes: 1) Distribution of *P. falciparum* genetic diversity in SSA and 2) Prevalence of *P. falciparum* polyclonal in SSA Secondary outcomes: Factors associated with *P. falciparum* genetic diversity and MOI in SSA
Settings:	All SSA malaria-affected countries
Time	2000-to-May 2023

## Results

**Fig. 1:**
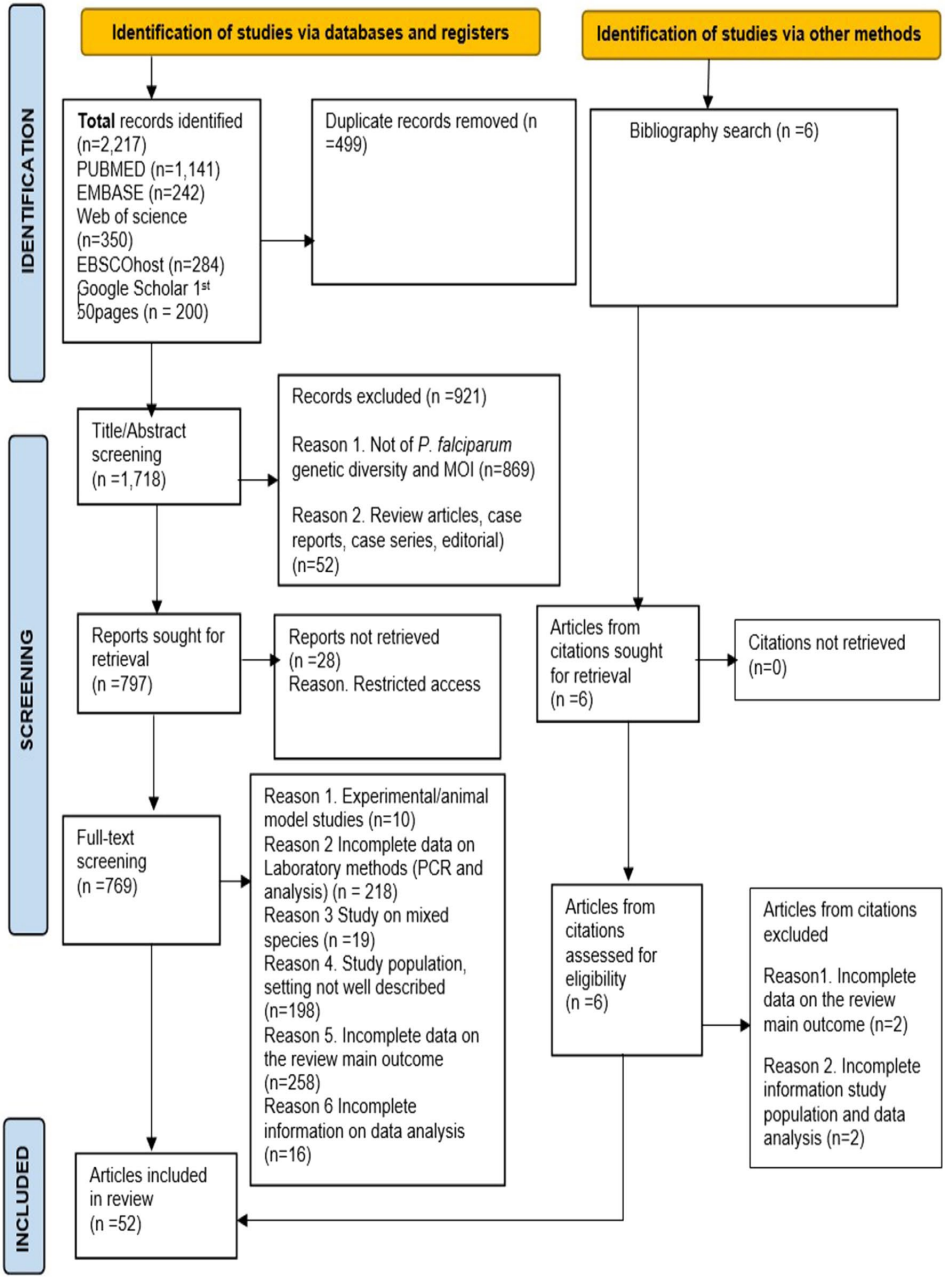
PRISMA. Flow diagram for identification of articles included in the review

A total of 1,718 articles were retrieved from the literature search, and an additional 6 articles were found through a search of the bibliographies of the identified articles. Of these, 52 articles met the inclusion criteria and were included in the review analysis (Fig. 1). The articles were from 23 of the 54 countries in SSA, covering a total of 11,640 genotyped parasite isolates from 9,062 symptomatic and 2,578 asymptomatic *Plasmodium falciparum* infections. Among the 52 articles, 39 (75%) employed cross-sectional study design while 13 (25%) utilized RCT/cohort study designs. A total of 23 studies enrolled both children and adults, while 22 studies enrolled only children, 2 studies enrolled only adults, and 5 studies did not specify the age group of their study population. The predominant genetic markers used to genotype parasites were the antigen-coding loci, especially *msp-1* and/or *msp-2*, in 76.9% (40/52) of the studies, followed by microsatellites markers only in 19.2% (10/52). In one study (1.92%; 1/52), both microsatellites and *msp-1* and/or *msp-2)* were used, while in another one study (1.92%; 1/52), genotyping of *Plasmodium falciparum* parasites involved the use of both *msp-1*, *msp-2*, and single nucleotide polymorphisms (SNPs) (Table 2).

**Table 2:** Summary of *P. falciparum* genetic diversity and MOI

First author and year	Country	Malaria clinical category	Age group	Number genotyped	Genotyped marker (s)	Genetic diversity							MOI	
						*msp-1* allelic frequencies				*msp-2* allelic frequencies		***glurp***		
						Mean He	%K1	%MAD20	%RO33	%IC/3D7	% FC27	***Number of haplotypes***	**Prevance of polyclonal infections**	**Mean MOI**
Cross sectional studies														
Chekol et al. 2022 [46]	Ethiopia	Asymptomatic	Children and adults	50	*msp-1 and msp-2*	0.23	46.5	65	37.2	90.7	62.8		50	1.56
Amoah et al.2021 [47]	Ghana	Asymptomatic	Children	119	Microsatellites	0.56–0.8							89.9	1.7
Touray et al. 2020 [16]	Kenya	Asymptomatic	Children	95	Microsatellites	0.81							79.69	3.39
Abukari et al. 2019 [48]	Ghana	Asymptomatic	Children and adults	160	*msp-2* and Microsatellites	0.67–0.69				68	24		45.-68	1.37
Mulenge et al. 2016 [49]	Kenya	Asymptomatic	NR	198	Microsatellites	0.84							80	2.8
Simpson et al. 2023 [17]	DRC	Asymptomatic and Symptomatic	Children	438	*msp-1* and *msp-2*		78	52	44	84	66		63	1.99
Gnagne et al. 2019 [50]	Cote d’Ivoire	Asymptomatic and Symptomatic	Children	282	*msp-1* and *msp-2*		81.6	53.4	57	84.3	72.2		85	3.16
Nabet et al. 2016 [51]	Mali	Asymptomatic and Symptomatic	Children and adults	156	Microsatellites	0.77							58.9	1.72
Apinjoh et al. 2015 [52]	Cameroon	Asymptomatic and symptomatic	Children and adults	151	*msp-1*		90.2(AS),71.6(SY)	52.5 (AS) 55.6 (SY)	42.6 (AS), 46.9(SY)				60	2.33
Gatei et al. 2015 [53]	Kenya	Asymptomatic and Symptomatic	Children	235	Microsatellites	0.77							89	2.65
Oyedeji et al. 2013 [54]	Nigeria	Asymptomatic and Symptomatic	Children	320	*msp-2*					38 (AS); 51 (SY)	59(AS); 45(SY)		62(AS); 60(SY)	2.0 (SY), 2.1 (AS)
Agomo et al. 2022 [55]	Nigeria	Symptomatic	Children	63	*msp-1*	0.64	52	35	87				63.1	1.98
Tadele et al. 2022 [19]	Ethiopia	Symptomatic	Children and adults	225	*msp-1* and *msp-2*	0.09	25.5 (HTS); 14.5(LTS)	87.2(HTS); 92.7 (LTS)	5 (HTS); 5.45 (LTS)	97.8 (HTS); 98.3 (LTS)	97.8 (HTS); 98.3 (LTS)		16.3	1.09
File et al. 2022 [56]	Ethiopia	Symptomatic	Children and adults	148	*msp-2*	0.49				31.8	27.7		40.5	1.4
Ajogbasile et al. 2021[57]	Nigeria	Symptomatic	Children	633	Microsatellites	0.804							67.1	NR
Gwarinda et al.2021[58]	S. Africa	Symptomatic	Adults	747	Microsatellites	0.74							66	2.13
Agaba et al. 2021 [59]	Uganda	Symptomatic	Children	85	Microsatellites								75.5	1.9
Oyedeji et al. 2020 [60]	Nigeria	Symptomatic	Children	93	*msp-2*					39.5	60.5		65.6	2.31
Papa Mze et al. 2020 [61]	Comoros	Symptomatic	NR	151	*msp-1*, *msp-2* and SNPS	0.715	55	15.2	3.2	38.7	47.4		80.6	1.43
Ndiaye et al. 2019 [62]	Senegal	Symptomatic	Children and adults	138	*msp-1 and msp-2*	0.394–0.637	71	31	38	83	48		36	2.56
Mohammed et al. 2019 [63]	Ethiopia	Symptomatic	Adults	118	*msp-1 and msp-2*		45.8	22	27.1	49.2	58.5		64.4	2.2
Nderu et al. 2019 [64]	Kenya	Symptomatic	NR	201	Microsatellites	0.76							51	1.8
Roh et al. 2019 [65]	Eswatini	Symptomatic	Children and adults	666	Microsatellites	0.75							67	2.2
Sane et al. 2019 [20]	Senegal	Symptomatic	Children and adults	71	*msp-1 and msp-2*		Micro 93.54; Submicro 87.5	Micro 60; Submicro54.83	Micro 41.93; Sub micro 22.5	Micro 61.29; Submicro 32.5	Micro 41.93, Submicr 10		35.48	*msp-1* 1.7–2.12 *msp-2* 1.03–1.60
Huang et al. 2018 [66]	Comoros	Symptomatic	Children	232	*msp-1 and msp-2*		51.8 (2006–2007); 41.8(2013–2016)	42.9 (2006–2007); 23.6 (2013–2016)	84.8 (2006–2007); 63.4 (2006–2007)	90.8(2006–2007); 37.1(2013–2016)	71.6(2006–2007); 91.1(2013–2016)		76.7(2006–2007); 28.3(2013–2016)	2.2
Chen et al. 2018 [22]	Equatorial Guinea	Symptomatic	Children and adults	181	*msp-1 and msp-2*		96.07	96.69	70.78	72.25	97.69		98.88	5.51
Niang et al. 2017 [67]	Senegal	Symptomatic	Children and adults	160	*msp-1 and msp-2*		89.37	87.5	62.5	96.25	29.37		*msp-1* 92.5; *msp-2* 28.75	2.23
Kolawole et al. 2016 [68]	Nigeria	Symptomatic	NR	50	*msp-1 msp-2 and glurp*	0.29–0.86	56	48	42	27.1	43.8	≥ 2	59	1.85
Mahdi Abdel Hamid et al. 2016 [69]	Sudan	Symptomatic	Children and adults	140	*msp-1, msp-2 and glurp*		38 (MM); 46 (SM)	55.5 (MM);42 (SM)	51 (MM); 50(SM)	76(MM); 62(SM)	77(MM); 68 (SM)		81	2.25
Kateera et al. 2016 [70]	Rwanda	Symptomatic	Children and adults	388	*msp-2*	0.413				70	68		44.6	1.73
Mawili-Mboumba, et al. 2015 [71]	Gabon	Symptomatic	Children	168	*msp-1*		65.5	20.8	55.9				50	1.72
Bouyou-Akotet et al. 2015 [72]	Gabon	Symptomatic	Children	112	*msp-1*		63.4	33.9	57.1				50	1.8
Ahmedou Salem et al. 2014 [73]	Mauritania	Symptomatic	Children and adults	113	*msp-1*		90	68.1	65.5				82.3	3.2
Oyebola et al. 2014 [37]	Nigeria	Symptomatic	Children and adults	536	*msp-1* and *msp-2*		60	50	45	55	62		50	1.4
Kiwuwa et al. 2013 [74]	Uganda	Symptomatic	Children	164	*msp-1, msp-2 and glurp*		82.9 (SM), 85.4(MM)	50 both SM and MM	48.8(SM); 37.8(MM)	92.7 Both SM and MM	86.8(SM) and 78(MM)	5 (360–1250 bp)	80.4	3.7 (SM) and 3.0 (MM)
Hamid et al. 2013 [75]	Sudan	Symptomatic	Children and adults	39	*msp-1 and msp-2*		31	39	41	59	64		62	1.93
Ogouyemi-Hounto et al. 2013 [36]	Benin	Symptomatic	Children	93	*msp-1 and msp-2*		85.2	67	82.9	81.5	98.9		89.4	3.8
Olasehinde et al. 2012 [76]	Nigeria	Symptomatic	NR	100	*msp-1, msp-2 and glurp*		68	40	20	76	56	5 (700–900 bp)	NR	1.87
Awaga et al. 2012[77]	Togo	Symptomatic	Children	309	*msp-1* and *msp-2*		54.58	25	20.42	48.29	51.71		26.16(MM)28.67 (SM)	3
RCT/Cohort studies														
Mohammed et al. 2021 [78]	Ethiopia	Symptomatic	Children and adults	41	*msp-2*	0.5				34.1	22		40.4	1.2
Abamecha et al. 2020 [79]	Ethiopia	Symptomatic	Children and adults	80	*msp-1* and *msp-2*	0.43–0.85	20.8	4.2	4.2	15.9	26.1		80	3.2
Sondo et al. 2020 [24]	Burkina Faso	Symptomatic	Children and adults	724	*msp-1* and *msp-2*		51	27	22	58	42		NR	2.73
Singana et al.2019 [80]	DRC	Symptomatic	Children	71	*msp-1* and *msp-2*	0.68–0.93	41	35	24	49.6	48.8		86	2.64
Mohammed et al. 2018 [81]	Ethiopia	Symptomatic	Children and adults	90	*msp-1, msp-2* and *glurp*	0.2–0.82	41.1	47.7	35.7	76	77	9 (301–800 bp)	70	2.6
Somé et al. 2018 [21]	Burkina Faso	Symptomatic	Children	228	*msp-1* and *msp-2*		77.4	41.3	36	93.1	41.3		61.9	1.95
Mohammed et al. 2017 [82]	Ethiopia	Symptomatic	Children and adults	92	*msp-2*	0.66				51	49		76	2.8
Kidima, W., et al.2015 [83]	Tanzania	Symptomatic	Children	82	*msp-2*					48.1	27.3		50	1.4
Mohammed, H., et al.2015 [84]	Ethiopia	Symptomatic	Children and adults	88	*msp-1* and *msp-2*	0.54–0.79	33.9	8.5	15.2	21.5	10.3		60	1.8
Ibara-Okabande 2012 [85]	DRC	Symptomatic	Children	52	*msp-2*					43	57		54	1.78
Sumari et al. 2010 [86]	Tanzania	Symptomatic	Children	300	*msp-2*					52.5	47.5		60.6	1.45
Aubouy et al. 2003 [87]	Gabon	Symptomatic	Children	52	*msp-1* and *msp-2*		90.4	63.5	36.5	82.7	65.4		NR	4
Peyerl-Hoffman et al. 2001[88]	Uganda	Symptomatic	Children and adults	225	*msp-1* and *msp-2*		81.1	41.3	35.5	66.7	55.6		71	2.4

AS-Asymptomatic, DRC – Democratic Republic of Congo, *glurp*-Glutamate rich protein, He- Expected heterozygosity, HTS-High malaria transmission setting, LTS- Low malaria transmission setting, Micro-Microscopic parasitemia, MM-Mild malaria, MOI- multiplicity of infection, *msp*-Merozoite surface protein, NR-Not reported, Ref-Reference, RCT-Randomized controlled trial, Sub micro-submicroscopic parasitemia, SM-severe malaria, SY-Symptomatic

### *Plasmodium falciparum* genetic diversity in SSA

Across studies, *P. falciparum* genetic diversity, primarily assessed using antigen-coding loci (*msp-1, msp-2,* and *glurp*), and microsatellites, was reported using either allele frequency, mean expected heterozygosity, or both. The frequencies of *msp-1* alleles (K1, MAD20, and RO33) were 20.8%, 4.2%, and 4.2%, respectively, in Ethiopia, a country with moderate malaria transmission in East Africa [79]. In high malaria transmission areas of Equatorial Guinea in West Africa, these same alleles had substantially higher frequencies of 96.07%, 96.09%, and 70.78%, respectively [22]. The frequency of the *msp-2* gene I/C 3D7 allele ranged from 15.9% to 98.3% in Ethiopia [19, 79], while the FC27 allele frequency ranged from 10.3% in Ethiopia [84] to 98.9% in high malaria transmission areas in Benin [36]. Meanwhile, the frequency of *glurp* ranged from 39.53% among symptomatic individuals in a high malaria transmission setting in Nigeria [68] to 97.6% among severe malaria cases living in malaria-moderate areas in Uganda [74] (Table 2).

The overall pooled allele frequencies of *msp-1* alleles K1, MAD20, and RO33 were 61%, 44%, and 40%, respectively, while the overall polled allele frequencies of *msp-2* I/C 3D7 and FC27 alleles were 61% and 55%, respectively, across reviewed studies. Across regions, the pooled allele frequencies of *msp-1* alleles K1, MAD20, and RO33 were 46%, 42%, and 31%, respectively, in East Africa; 74%, 51%, and 48%, respectively, in Central Africa; and 67%, 43%, and 44%, respectively, in West Africa. In comparison, the pooled allele frequencies of the *msp-2* I/C 3D7 and FC27 alleles were 60% and 55%, respectively, in East Africa, 67% in Central Africa, and 62% and 50%, respectively, in West Africa. For *glurp*, the overall pooled allele frequency was 66%, with a pooled frequency of 90% and 70% in East and West African regions, respectively. The only two reviewed studies from Southern Africa [58, 65] studied parasite genetic diversity and MOI using only microsatellites and not *msp-1, msp-2*, or *glurp* (Table 3).

**Table 3:** Pooled proportions of K1, MAD20, RO33, IC/3D7, FC27 and *glurp* alleles across studies

Gene family	Alleles	East Africa		Central Africa		West Africa		All regions	
		Freq (%)	95% CI	Freq (%)	95% CI	Freq (%)	95% CI	Freq (%)	95% CI
*msp-1*	K1	46	31–60	74	60–88	67	56–78	61	53–69
	MAD20	42	23–60	51	22–81	43	34–51	44	32–56
	RO33	31	17–45	48	35–60	44	33–58	40	32–48
*msp-2*	IC 3D7	60	48–72	67	52–82	62	51–72	61	54–69
	FC 27	55	42–68	67	45–90	50	33–68	55	46–64
*glurp*		90	86–93	–	–	70	63–77	66	40–92

**Fig. 2:**
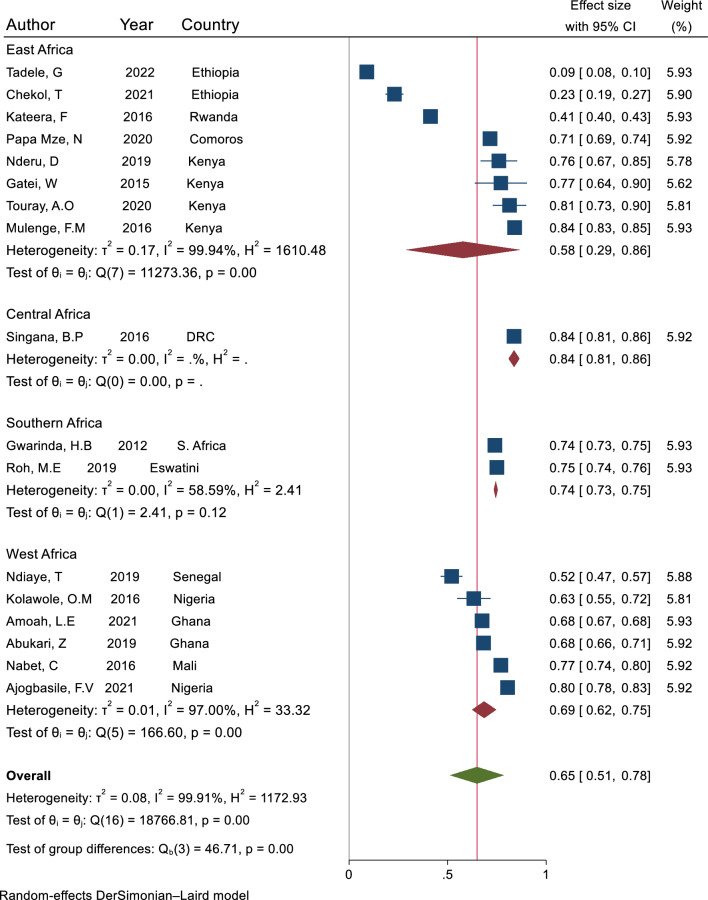
Forest plot representing the pooled mean expected heterozygosity of *P. falciparum* infection across 17 studies that reported measures of dispersion (CI and SD) for mean expected heterozygosity in malaria-affected countries in SSA, sorted by region

Based on expected heterozygosity, the mean expected heterozygosity was 0.09 in Ethiopia [19], a country with moderate malaria transmission in East Africa, and 0.93 in the DRC [80], a country with high malaria transmission in Central Africa (Table 2). The overall pooled mean expected heterozygosity across all studies was 0.65 (95% CI: 0.51–0.78). Across regions, the pooled mean expected heterozygosity was 0.58 (95% CI: 0.29–0.86), 0.84 (95% CI: 0.81–0.86), 0.74 (95% CI: 0.73–0.75), and 0.69 (95% CI: 0.62–0.75) in East, Central, Southern, and West African regions, respectively (Fig. 2).

Each blue square bar indicates the estimated mean expected heterozygosity in one study, and the lines through the square represent the confidence interval around the estimate. The red diamond symbols represent the pooled mean expected heterozygosity in each region, while the green diamond symbol represents the overall pooled mean expected heterozygosity across all regions. The x-axis represents the scale for mean expected heterozygosity which ranges between 0 to 1.

### *Plasmodium falciparum* MOI across malaria-affected countries in SSA

**Fig. 3:**
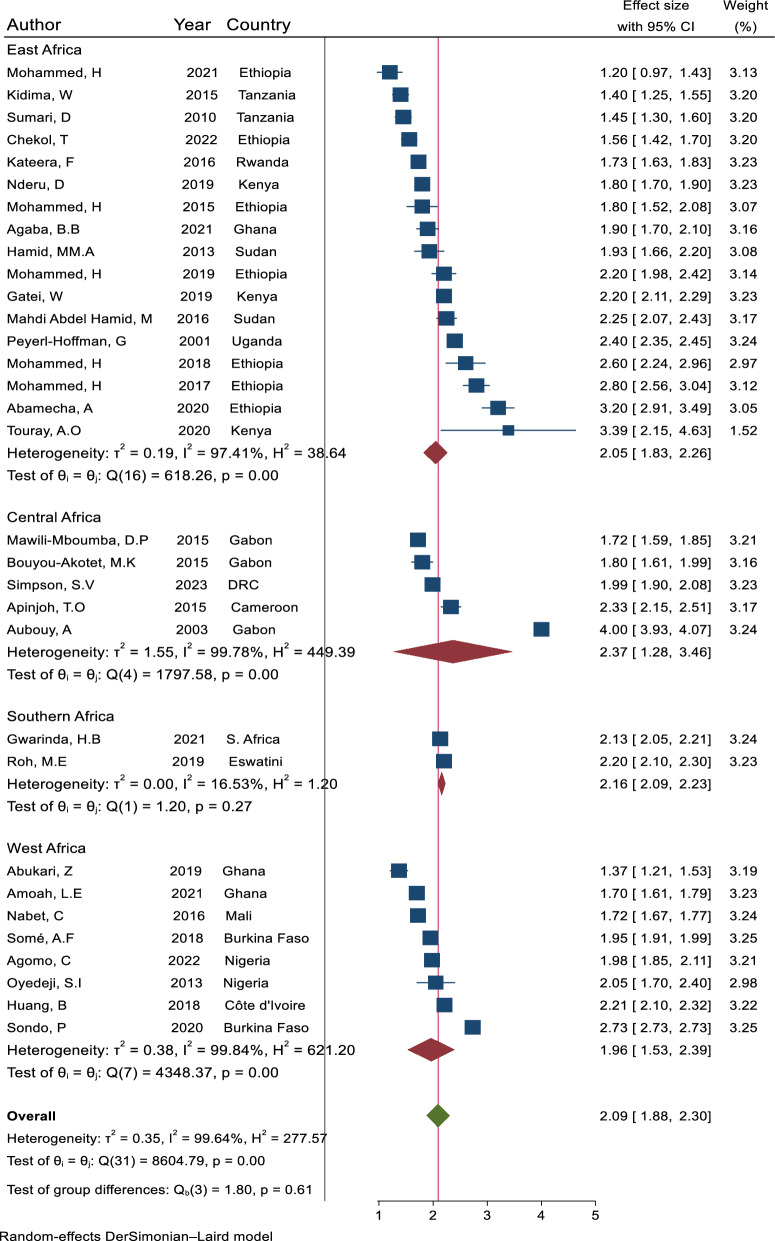
Forest plot representing the pooled mean MOI of *P. falciparum* infection across 32 studies that reported measures of dispersion (CI and SD) for mean MOI in malaria-affected countries in SSA, sorted by region. Each blue square bar indicates the estimated mean *P. falciparum* MOI in one study, and the lines through the square represent the confidence interval around the estimate. The red diamond symbols represent the pooled mean *P. falciparum* MOI in each region, while the green diamond symbol represents the overall pooled *P. falciparum* mean MOI across all regions. The x-axis represents the scale for mean MOI

*Plasmodium falciparum* MOI, defined as the number of distinct parasite genotypes co-existing within a given infection. *Plasmodium falciparum* MOI was reported using mean MOI and or prevalence of polyclonal infection. The mean MOI ranged from 1.09 in Ethiopia in East Africa [19] to 5.51 in Equatorial Guinea in West Africa [22] (Table 2). The overall pooled mean MOI across studies was 2.09 (95% CI: 1.88–2.30). Across regions, the pooled mean MOI was 2.05 (95% CI: 1.83–2.26), 2.37 (95% CI: 1.28–3.46), 2.16 (95% CI: 2.09–2.23), and 1.96 (95% CI: 1.53–2.39) in East, Central, Southern, and West African regions, respectively (Fig. 3).

**Fig. 4:**
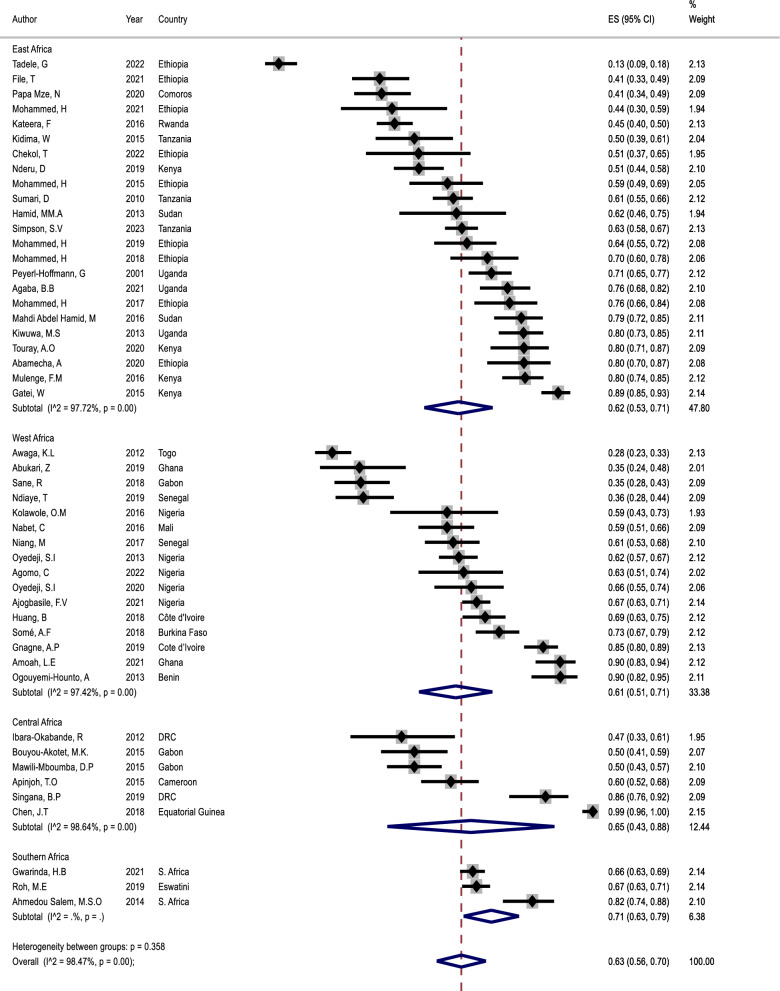
Forest plot representing the pooled prevalence *P. falciparum* polyclonal infections reported by 48 studies from malaria-affected countries in SSA, sorted by region. Each gray square bar with a black dot indicates the estimated prevalence of *P. falciparum* polyclonal infections in one study, and the lines through the square represent the confidence interval around the estimate. The diamond symbol represents the pooled prevalence of *P. falciparum* polyclonal infections

The prevalence of polyclonal infections ranged from 16.3% in Ethiopia in East Africa [19] to 98% in Equatorial Guinea in Central Africa [22] to 98% in Equatorial Guinea in Central Africa [27] (Table 2). The overall pooled prevalence of *Plasmodium falciparum* polyclonal infections was 63% (95% CI 56–70) across all studies. Across the regions, the pooled prevalence of polyclonal infections was 62% (95% CI: 53–71), 61% (95% CI: 51–71), 65% (95% CI: 43–88), and 71% (95% CI: 63–79) in East, West, Central, and Southern Africa regions, respectively (Fig. 4).

### Factors associated with *Plasmodium falciparum* genetic diversity and MOI across SSA

In three studies [24, 72, 88], a positive association between patient age and parasite density with *Plasmodium falciparum* genetic diversity and MOI was observed; however, this association was not consistent across other studies [36, 69, 79]. Some studies indicated an association between *Plasmodium falciparum* genetic diversity and MOI with the use of chemotherapy to suppress malaria infections. For instance, in a study by Huang, B et al*.* [66], a decrease in genetic diversity was found over a 10-year period following the introduction of artemisinin-based combination therapy (ACT) in an island population, with a 28% decrease for *msp-1* (from 32 to 23) and *msp-2* (from 29 to 21). MOI declined from 3.11 to 1.63 for *msp-1* and from 2.75 to 1.35 for *msp-2*. The prevalence of polyclonal infection for *msp-1* declined from 76.7% to 29.1% (P < 0.01), and for *msp-2*, it declined from 62.4% to 28.3% (P < 0.01).

Similarly, a study by Tadele et al*.* [19] reported a decline in *Plasmodium falciparum* genetic diversity and MOI. Variations in *Plasmodium falciparum* genetic diversity and MOI were found in both rural and urban settings. MOI was higher in rural than in urban settings; for instance, the mean MOI for rural versus urban areas was 1.88 versus 1.55, while the prevalence of polyclonal infection was 42.2% versus 57.7% (p = 0.04) [71]. However, in a study conducted in an urban setting in Uganda [74], the mean MOI values were even higher (3.0 to 3.7 for severe and mild malaria cases, respectively p = 0.002) than those observed in rural areas elsewhere. High *Plasmodium falciparum* genetic diversity and MOI were also reported among both symptomatic [72, 74, 89] and asymptomatic malaria cases [16, 90]. Furthermore, a positive association between the genetic diversity and MOI of *Plasmodium falciparum* with malaria transmission settings, showing higher values in areas with high malaria transmission and lower values in those with low malaria transmission (2.13 in high and 1.29 in low malaria transmission; p < 0.0001) has been reported [70]. Meanwhile, the expected heterozygosity was high (0.49 to 0.62) and low (0.26 to 0.28) in high and low malaria transmission settings, respectively. However, this relationship was not observed elsewhere reported [37].

### Subgroup analysis of *Plasmodium falciparum* genetic diversity and MOI based on malaria clinical category and the genotyped markers

Subgroup analysis of genetic diversity and MOI was conducted using mean expected heterozygosity and mean MOI, respectively. Considering patient phenotype, the pooled mean expected heterozygosity was 0.64 (95% CI 0.515–0.78) in studies that enrolled only individuals with asymptomatic infection and 0.63 (0.42–0.83) in those that enrolled only individuals with symptomatic infection. However, the pooled mean expected heterozygosity was 0.77 (0.75–0.79) in studies that enrolled individuals with either asymptomatic or symptomatic *Plasmodium falciparum* infections.

Based on antigen-coding loci, *msp-1*, and/or *msp-2* genotypes, the pooled *Plasmodium falciparum* mean expected heterozygosity was 0.49 (95% CI 0.24–0.74). In comparison, it was 0.76 (95% CI 0.72–0.79) based on microsatellite markers. The pooled mean MOI was 1.90 (95% CI 1.50–2.30) in studies enrolling asymptomatic individuals, 2.16 (95% CI 1.92–2.41) in studies enrolling symptomatic individuals, and 1.85 (95% CI 1.59–2.11) in studies enrolling both asymptomatic and symptomatic cases. In studies using antigen-coding loci *msp-1* and/or *msp-2* only, the pooled mean MOI was 2.14 (95% CI 1.91–2.38), while in studies using microsatellites, it was 1.63 (95% CI 1.11–2.15).

### *Plasmodium falciparum* heterogeneity in the included studies

Studies were combined and assessed for heterogeneity. Based on mean expected heterozygosity, there was high heterogeneity among studies enrolling asymptomatic individuals (I^2^ = 99.61%, P < 0.001) and among studies enrolling individuals with symptomatic malaria (I^2^ = 99.94%, P < 0.001). Similarly, a high level of heterogeneity was observed among studies using antigen-coding loci, namely *msp-1* and *msp-2* (I^2^ = 99.87%, P < 0.001), as well as microsatellites (I^2^ = 98.28%, P < 0.001). Regarding mean MOI, heterogeneity was high in studies enrolling individuals with asymptomatic malaria (I^2^ = 98.64%, P < 0.001), symptomatic malaria (I^2^ = 99.60%, P < 0.001), and both asymptomatic and symptomatic cases (I^2^ = 96.20%, P < 0.001). A high level of heterogeneity was also observed across the genotyped markers, including antigen coding loci, *msp-1*, and *msp-2* (I^2^ = 99.619%, P < 0.001), and microsatellites (I^2^ = 96.52%, P < 0.001) (Additional file 3).

### Risk of bias in the included studies

Methodological quality and reporting bias were identified as high in 28.8% (15/52) of the studies. There was a potential for selection bias, as successful genotyping was reported to be < 90% in 11.5% (6/52) of the reviewed articles. Additionally, detection bias related to the assessment of confounding factors was noted in 36.5% (19/52) of the included articles (Additional file 4).

### Publication bias assessment

Visual inspection of the funnel plots obtained using mean expected heterozygosity and mean MOI revealed an asymmetrical distribution of estimates from the middle line (Additional file 5). Egger’s statistical test showed a coefficient of 2.16, z = 0.54, and P = 0.59 for mean expected heterozygosity, and a coefficient of 1.65, z = 1.28, and P = 0.2 for mean MOI.

## Discussion

The systematic review covered studies investigating *Plasmodium falciparum* genetic diversity and MOI in malaria-affected countries in SSA. Study findings indicate substantial genetic diversity and MOI among parasites circulating in SSA. The substantial regional variation in parasite genetic diversity and MOI identified in this current study likely reflects differences in regional malaria transmission intensity. This suggests that these markers may be useful in evaluating malaria transmission patterns and the effectiveness of control interventions.

Parasite genetic diversity exhibited variations across regions, with *msp-1* (K1, MAD20, and RO33) and *msp-2* (I/C3D7 and FC27) alleles showing different frequencies in different regions. The finding that high *Plasmodium falciparum* genetic diversity was reported in both high [51] and low [65] malaria transmission areas in SSA is interesting. Parasite genetic diversity results from genetic recombination in the mosquito [6, 91], and is more likely in areas with high local malaria transmission intensity. The high parasite genetic diversity in some areas is therefore a cause for concern because it may indicate ongoing transmission despite the intensification of malaria control measures [53]. Nonetheless, there are areas with low *Plasmodium falciparum* genetic diversity, indicating that malaria control and surveillance efforts should be tailored accordingly.

*Plasmodium falciparum* mean MOI also exhibited wide variations across regions, ranging from 1.09 to 5.51, with an overall pooled mean MOI of 2.09. Meanwhile, the prevalence of polyclonal infection also varied significantly, ranging from 16.3% to 98%, with an overall pooled prevalence of 63% across studies. Previous reports have documented wide variations in *Plasmodium falciparum* mean MOI (ranging from 1 to 6.1) and the percentage prevalence of polyclonal infections (ranging from 0 to 96%) [25]. High mean MOI and the presence of polyclonal infections serve as key indicators of high malaria transmission intensity [16, 70]. These factors are influenced by increased vector populations, promoting either superinfection or the concurrent transmission of unrelated parasite genotypes [92]. The variations across regions suggest differences in malaria transmission patterns across SSA, emphasizing the need for modifications in malaria vector control and the implementation of customized regional malaria control measures.

The review identified several factors associated with *Plasmodium falciparum* genetic diversity and MOI, including parasite density, the clinical category of malaria infection, patient age, malaria control interventions, and malaria transmission intensity [16, 19, 24, 48, 70, 72, 89]. These findings extend those from previous studies that reported a positive correlation between parasite density and parasite genetic diversity/MOI [70, 75]. Higher parasite density increases the likelihood of carrying distinct parasite genotypes [70], while an increase in age enhances immunity to malaria [93] Additionally, *Plasmodium falciparum* genetic diversity and MOI were found to be higher in rural settings [71], although other reports indicated higher genetic diversity in urban settings [74]. Low parasite genetic diversity and MOI have been reported in areas of Ethiopia, suggesting the effectiveness of malaria control interventions [19].

High *Plasmodium falciparum* genetic diversity and MOI were reported among both symptomatic [72, 74, 89] and asymptomatic malaria cases [16, 48, 90]. The occurrence of multiple *Plasmodium falciparum* infections could pose a challenge to parasite elimination efforts [16] due to its positive association with antimalarial drug failure [94]. Asymptomatic infection is typically characterized by low parasitaemia [95] and high MOI [38]. Asymptomatic individuals with low parasitaemia often remain undetected, thus forming a reservoir for malaria transmission and its spread [96]. The variability in parasite genetic diversity infection profiles has implications for treatment strategies, as well as the efficacy of antimalarial drugs.

*msp-1* and *msp-2* are commonly used genetic markers for assessing *Plasmodium falciparum* genetic diversity and MOI. Studies exclusively employing antigen-coding loci (*msp-1* and/or *msp-2*) reported a higher pooled mean MOI (2.14), while those utilizing microsatellites showed a lower pooled mean MOI (1.63). The abundance and high polymorphism of microsatellites make them more suitable for estimating MOI compared to *msp-1, msp-2*, and *glurp*, which are relatively fewer and exhibit lower levels of polymorphism [13]. Moreover, *msp-1, msp-2*, and *glurp* are susceptible to immune selection [97]. Another limitation arises from significant size variations among *msp-1, msp-2*, and *glurp* alleles, potentially introducing bias in amplification efficiency. In cases of multiclonal infections, this bias may result in the preferential amplification of shorter fragments, leading to the loss of longer alleles [14, 15]. This emphasizes the importance of employing advanced tools such as microsatellite analysis and whole-genome sequencing for accurately assessing *Plasmodium falciparum* genetic diversity.

### Implications for future research and policy

The substantial variations in *Plasmodium falciparum* genetic diversity and MOI in SSA necessitates continuous genomic surveillance in different malaria transmission settings. Current research predominantly focuses on symptomatic malaria infections in children utilising *msp-1* and *msp-2* genetic markers for assessing genetic diversity and MOI. Future studies should broaden their focus to include both adults and children across different malaria transmission contexts. Incorporation of advanced tools like microsatellite and whole-genome sequencing, is crucial for accurate assessments of parasite genetic diversity.

### Strengths of the study

The review focused on peer-reviewed articles published over an extended period of time to adequately appreciate the genetic diversity and MOI of *Plasmodium falciparum* parasites circulation in SSA, an area which contributes over 95% of global malaria cases. The review focused on genetic markers (*msp-1, msp-2, glurp*, and microsatellites) that are more common and readily available in resource-limited settings in SSA. The review was conducted following standard PRISMA-P review guidelines to enhance the reliability of the findings.

### Limitations of the study

The present study has several limitations. Firstly, reliance on peer-reviewed published articles may have introduced potential publication bias. Secondly, studies that did not explicitly mention *Plasmodium falciparum* genetic diversity and/or MOI in the title may have been missed. Additionally, the geographical coverage of articles was not comprehensive, as they did not encompass all countries in SSA, thereby impacting the generalizability of findings within the region. For instance, only two of the reviewed studies originated from Southern Africa [58, 65], focusing solely on microsatellites rather than *msp-1, msp-2*, and *glurp*, which limits inferences about marker distribution in this region. Furthermore, the exclusion of studies lacking measures of dispersion (CI and SD) affected the meta-analysis of mean expected heterozygosity and mean MOI.

## Conclusion

This systematic review reveals considerable variations in *Plasmodium falciparum* genetic diversity and MOI across malaria-affected countries in SSA. Despite control efforts, the high observed parasite genetic diversity and MOI emphasize the necessity for customized, region-specific malaria control strategies, and continuous surveillance.

### Supplementary Information

**Additional file 1.** Search strategy.

**Additional file 2. **Screening criteria.

**Additional file 3.** Subgroup and Heterogeneity analysis.

**Additional file 4. **RoB assessment.

**Additional file 5. **Publication bias assessment

## Data Availability

The datasets generated and/or analysed in this review are available from the corresponding author upon reasonable request.

## Abbreviations

GRADE: Grading of Recommendations, Assessment and Development and Evaluations
HRP2: Histidine rich protein 2
JBI: Joanna Briggs Institute’s
MOI: Multiplicity of infection
MSP: Merozoite surface protein
NR: Not reported
PCR: Polymerase chain reaction
PRISMA-P: Reporting Items for Systematic Review and Meta-Analysis for Protocols
RoB: Risk of bias
STREGA: STrengthening the REporting of Genetic Association Studies
STROBE: Strengthening the Reporting of Observational Studies in Epidemiology
