# Supplementary material for: Plasmodium falciparum genetic diversity and multiplicity of infection based on msp-1, msp-2, glurp and microsatellite genetic markers in sub-Saharan Africa: a systematic review and meta-analysis
Source: Malar J. 2024 Apr 8;23:97. doi: 10.1186/s12936-024-04925-y (PMC11000358; doi:10.1186/s12936-024-04925-y)
Supplement: Supplementary file 1 — Additional file 1. Search strategy. [file 12936_2024_4925_MOESM1_ESM.doc]

***Plasmodium falciparum* genetic diversity and multiplicity of infection based on *msp-1*, *msp-2*, *glurp* and microsatellite genetic markers in sub-Saharan Africa: a systematic review and meta-analysis**

UPDATED SEARCH STRATEGY-26 MAY-6 JUNE 2023

| **DATABASE / DATE SEARCHED** | **SEARCH STRING** | **RESULTS** |
| --- | --- | --- |
| **PUBMED – 6 JUNE 2023** |  |  |
| 1 | IN TW: Plasmodium falciparum OR P. falciparum OR Genetic diversity of Plasmodium falciparum OR Plasmodium falciparum genotype* OR Plasmodium falciparum Multiplicity of infection OR Plasmodium falciparum Complexity of infection | 46,601 |
| 2. | (Malaria-endemic countr* OR malaria-endemic region* OR malaria-endemic area* OR malaria endemic setting*) AND (Western Sahara OR OR Central Africa OR West Africa OR Western Africa OR East Africa OR Eastern Africa OR Southern Africa OR Southern African OR sub Saharan Africa OR Africa South of the Sahara) | 324,130 |
| 3. | 1 AND 2 | 1,251 |
| 4 | Filters: 2000-2023 | 1,141 |
|  |  |  |
| EMBASE / 26 MAY 2023  1 | (Plasmodium falciparum or P falciparum or Genetic diversity of Plasmodium falciparum or Plasmodium falciparum genotype* or Plasmodium falciparum Multiplicity of infection or Plasmodium falciparum Complexity of infection) | 59,937 |
| 2 | (Malaria-endemic countr* OR malaria-endemic region* OR malaria-endemic area* OR malaria endemic setting*) AND (Western Sahara OR OR Central Africa OR West Africa OR Western Africa OR East Africa OR Eastern Africa OR Southern Africa OR Southern African OR sub Saharan Africa OR Africa South of the Sahara) | 959 |
| 3 | 1 OR 2 | 412 |
| 4 | Filter: from 2000 - 2023 | 242 |
|  |  |  |
| Web of Science /26 MAY 2023  1 | TS=(Plasmodium falciparum or P falciparum or Genetic diversity of Plasmodium falciparum or Plasmodium falciparum genotype* or Plasmodium falciparum Multiplicity of infection or Plasmodium falciparum Complexity of infection) | 56,006 |
| 2 | (Malaria-endemic countr* OR malaria-endemic region* OR malaria-endemic area* OR malaria endemic setting*) AND (Western Sahara OR OR Central Africa OR West Africa OR Western Africa OR East Africa OR Eastern Africa OR Southern Africa OR Southern African OR sub Saharan Africa OR Africa South of the Sahara) | 1,982 |
| 3 | 1 AND 2 | 1,113 |
| 4 | Filter: 2000-2023 | 350 |
|  |  |  |
| ENSCOhost / 26 MAY 2023  S1 | Plasmodium falciparum or Plasmodium falciparum genotype* or Plasmodium falciparum Multiplicity of infection or Plasmodium falciparum Complexity of infection | 20,055 |
| S2 | (Malaria-endemic countr* OR malaria-endemic region* OR malaria-endemic area* OR malaria endemic setting*) AND (Western Sahara OR OR Central Africa OR West Africa OR Western Africa OR East Africa OR Eastern Africa OR Southern Africa OR Southern African OR sub Saharan Africa OR Africa South of the Sahara) | 1,550 |
| S3 | 1 AND 2 | 642 |
| S4 | Filter: 2000-2023 | 284 |
| GOOGLE SCHOLAR / 6 JUNE 2023 | (Plasmodium falciparum OR Genetic diversity of Plasmodium falciparum OR Plasmodium falciparum genotype* OR Plasmodium falciparum Complexity of infection) AND (malaria endemic countries) AND (Sub Saharan Africa) | 6,400 (1^st^ 50 pages) = 200 |
| TOTAL |  | 2,217 |
| DUPLICATES |  | 499 |
| BALANCE FOR SCREENING |  | 1,718 |
