# Supplementary material for: Plasmodium falciparum genetic diversity and multiplicity of infection based on msp-1, msp-2, glurp and microsatellite genetic markers in sub-Saharan Africa: a systematic review and meta-analysis
Source: Malar J. 2024 Apr 8;23:97. doi: 10.1186/s12936-024-04925-y (PMC11000358; doi:10.1186/s12936-024-04925-y)
Supplement: Supplementary file 3 — Additional file 3. Subgroup and Heterogeneity analysis. [file 12936_2024_4925_MOESM3_ESM.doc]

***Plasmodium falciparum* genetic diversity and multiplicity of infection based on *msp-1*, *msp-2*, *glurp* and microsatellite genetic markers in sub-Saharan Africa: a systematic review and meta-analysis**

Met-analysis (Subgroup and heterogeneity analysis)

1. Based on mean expected heterozygosity (He)

1. Based on the mean multiplicity of infection (MOI)

Key

Malaria clinical-y

1. Asymptomatic malaria infection
2. Symptomatic malaria infection
3. Both a symptomatic and symptomatic malaria infection

Genotyped markers

1. Antigen coding loci such as *msp-1, msp-2* and *glurp*
2. Microsatellites
