# Supplementary material for: Plasmodium falciparum genetic diversity and multiplicity of infection based on msp-1, msp-2, glurp and microsatellite genetic markers in sub-Saharan Africa: a systematic review and meta-analysis
Source: Malar J. 2024 Apr 8;23:97. doi: 10.1186/s12936-024-04925-y (PMC11000358; doi:10.1186/s12936-024-04925-y)
Supplement: Supplementary file 4 — Additional file 4. RoB assessment. [file 12936_2024_4925_MOESM4_ESM.doc]

***Plasmodium falciparum* genetic diversity and multiplicity of infection based on *msp-1*, *msp-2*, *glurp* and microsatellite genetic markers in sub-Saharan Africa: a systematic review and meta-analysis**

Risk of bias (RoB) assessment for included studies

| Author, year | Selection bias due to sample size or sampling criteria | Selection bias due to the proportion of successful genotyping (< 90%) | Selection bias due to baseline characteristics (study participants and settings) or not described | Detection bias due to unreliable measurement of exposure | Detection bias due to the reliability of genetic markers and measurement tools used for the outcome | Detection bias due to confounding factors | Bias due to method of statistical analysis used for overall outcome | Bias due to method of data analysis used for associated factors | Aggregate assessment per article |
| --- | --- | --- | --- | --- | --- | --- | --- | --- | --- |
| Oyedeji, 2013 | – | ? | – | - | + | ? | – | + | High |
| Singana, 2019 | - | - | - | - | - | ? | - | - | Low |
| Okabande, 2012 | - | - | ? | - | + | ? | - | - | Moderate |
| Salem, 2014 | - | ? | - | - | + | - | – | - | Moderate |
| Olasehinde, 2012 | - | ? | – | – | – | ? | – | + | Moderate |
| Kolawole,2016 | + | – | ? | - | – | ? | – | + | High |
| Ndiaye,2019 | + | ? | - | – | – | ? | - | ? | High |
| Kidima, 2015 | - | + | - | - | + | ? | - | + | High |
| Sane, 2018 | - | - | - | - | – | - | - | - | Low |
| Mohammed,2017 | – | ? | – | – | + | ? | – | + | High |
| Abukari, 2019 | – | - | – | - | - | ? | – | - | Low |
| Kiwuwa, 2013 | - | - | – | - | – | ? | - | + | Low |
| Mboumba, 2015 | – | ? | - | - | + | + | ? | – | High |
| Hamid, 2013 | – | – | - | – | – | ? | – | - | Low |
| Chekol, 2022 | – | + | – | – | - | - | – | - | Low |
| Hounto, 2013 | - | – | – | ? | – | + | ? | + | High |
| Akotet, 2015 | - | - | ? | ? | + | - | – | - | Moderate |
| Apinjoh, 2015 | - | - | - | - | - | - | ? | - | Low |
| Amoah, 2021 | - | - | – | - | - | ? | - | - | Low |
| Mohammed, 2015 | - | - | - | - | - | + | - | + | Moderate |
| Nabet, 2016 | - | – | - | ? | - | – | ? | + | Moderate |
| Huang, 2018 | - | ? | - | - | ? | – | + | + | High |
| Mohammed, 2018 | - | - | - | - | - | + | - | + | Moderate |
| Chen, 2018 | – | – | – | - | + | ? | – | - | Low |
| Mohammed, 2019 | - | - | + | - | - | - | - | - | low |
| Abamecha, 2020 | - | - | - | - | - | - | - | - | low |
| Oyedeji, 2020 | + | - | ? | - | + | ? | ? | ? | High |
| Mohammed, 2021 | ? | + | - | - | + | ? | - | - | High |
| Ajogbasile, 2021 | - | - | - | - | - | - | ? | ? | Low |
| File, 2021 | - | - | - | - | + | ? | - | - | Moderate |
| Hamid, 2016 | - | - | - | - | - | ? | - | - | low |
| Niang, 2017 | - | ? | - | - | - | ? | - | - | Low |
| Somé, 2018 | - | - | - | - | - | - | - | + | low |
| Sondo, 2020 | - | ? | - | - | - | - | - | - | Low |
| Touray, 2020 | - | - | - | - | - | ? | - | - | low |
| Awaga, 2012 | - | - | - | - | - | - | - | ? | Low |
| Nderu, 2019 | - | - | ? | - | ? | + | - | - | moderate |
| Aubouy, 2003 | ? | ? | - |  | - | ? | - | + | Moderate |
| Agomo, 2022 | + | - | ? | - | + | ? | - | - | High |
| Sumari, 2010 | - | ? | - | - | + | ? | - | - | Moderate |
| Gnagne, 2019 | - | ? | - | - | - | - | - | - | low |
| Mulenge, 2016 | ? | - | - | - | - | + | - | + | Moderate |
| Roh, 2019 | - | + | - | - | - | - | - | - | Low |
| Gatei, 2015 | - | ? | - | - | - | ? | -- | - | Low |
| Mze, 2020 | - | - | - | ? | - | ? | - | ? | Moderate |
| Gwarinda, 2021 | - | + | - | ? | - | - | - | - | Moderate |
| Agaba, 2021 | + | + | - | - | - | ? | - | ? | High |
| Kimenyi, 2022 | - | ? | - | - | + | ? | - | + | High |
| Tadele, 2022 | - | - | - | - | - | - | - | + | Low |
| Oyebola, 2014 | - | ? | - | - | - | + | ? | + | High |
| Hoffmann, 2001 | - | ? | - | - | - | - | - | - | Low |
| Kateera, 2016 | - | - | - | - | + | - | - | - | Low |
|  |  |  |  |  |  |  |  |  |  |

**Symbols used mean:* *Low risk (–), High risk (+), Moderate risk (?)*
