# Supplementary material for: Plasmodium falciparum genetic diversity and multiplicity of infection based on msp-1, msp-2, glurp and microsatellite genetic markers in sub-Saharan Africa: a systematic review and meta-analysis
Source: Malar J. 2024 Apr 8;23:97. doi: 10.1186/s12936-024-04925-y (PMC11000358; doi:10.1186/s12936-024-04925-y)
Supplement: Supplementary file 5 — Additional file 5. Publication bias assessment [file 12936_2024_4925_MOESM5_ESM.doc]

***Plasmodium falciparum* genetic diversity and multiplicity of infection based on *msp-1*, *msp-2*, *glurp* and microsatellite genetic markers in sub-Saharan Africa: a systematic review and meta-analysis**

1. Funnel plot based on mean expected heterozygosity

# Funnel plot based on Mean MOI

# 
